# Prevalence and incidence of noncystic fibrosis bronchiectasis among US adults in 2013

**DOI:** 10.1177/1479972317709649

**Published:** 2017-05-30

**Authors:** Derek Weycker, Gary L Hansen, Frederic D Seifer

**Affiliations:** 1 Policy Analysis Inc. (PAI), Brookline, MA, USA; 2 RespirTech, St. Paul, MN, USA; 3 St. Lawrence Health System, Potsdam, NY, USA

**Keywords:** Bronchiectasis, epidemiology, prevalence, incidence

## Abstract

Bronchiectasis is an incurable pulmonary disorder that is characterized pathologically by permanent bronchial dilatation and severe bronchial inflammation and clinically by chronic productive cough and recurrent infectious exacerbations; bronchiectasis often occurs in the presence of chronic obstructive pulmonary disease. It is widely believed that increasing use of high-resolution computed tomography has led to a marked rise in the number of persons with diagnosed bronchiectasis in current US clinical practice; up-to-date evidence, however, is lacking. Using a retrospective cohort design and health-care claims data (2009–2013), we estimated the prevalence of bronchiectasis (noncystic fibrosis)—based on narrow case-finding criteria—to be 139 cases per 100,000 persons, to be higher among women versus men (180 vs. 95 per 100 K), and to increase substantially with age (from 7 per 100 K to 812 per 100 K aged 18–34 years and ≥75 years, respectively); annual incidence was estimated to be 29 cases per 100,000 persons. Disease prevalence based on broad case-finding criteria was estimated to be 213 cases per 100,000 persons. The findings of this study suggest that between 340,000 and 522,000 adults were receiving treatment for bronchiectasis and that 70,000 adults were newly diagnosed with bronchiectasis, in 2013 US clinical practice. The findings of this study also suggest that bronchiectasis is much more common than previously reported (annual growth rate since 2001, 8%), presumably due—at least in part—to recent advances in, and increased use of, radiologic techniques. Additional research is needed to validate the findings of this study, to identify the reasons for increased prevalence, and to promote education about bronchiectasis nationally.

## At a Glance

Notwithstanding the seriousness of bronchiectasis, surprisingly little is known about the current epidemiology of this disorder among US adults, as the limited data that have been reported are now over a decade old and/or are focused on select subgroups of persons. The findings of this study suggest that the prevalence and incidence of bronchiectasis (noncystic fibrosis) is substantially higher than previously reported, that the diagnosis of bronchiectasis continues to increase in clinical practice (annual growth rate since 2001, 8%), and that diagnosed cases reflect a relatively small segment of the overall bronchiectasis population.

## Introduction

Bronchiectasis is an incurable pulmonary disorder characterized pathologically by permanent bronchial dilatation and severe bronchial inflammation and clinically by chronic productive cough and recurrent infectious exacerbations. Sometimes described as a disease, bronchiectasis is perhaps more appropriately thought of as the end result of a pathological process involving a vicious cycle of inflammation, recurrent infection, and bronchial wall damage that can occur in association with a variety of primary causes, including infectious, genetic, inflammatory, environmental, and allergic.^1,2^ While the etiology of bronchiectasis is unknown in many cases, an increasing percentage of patients in developed countries are diagnosed with specific underlying causes or contributing factors.^2^

Historically, the diagnosis and treatment of bronchiectasis have been challenging. With the advent of high-resolution computed tomography (HRCT) of the chest and advances in airway clearance in the early 1990s,^3^ non-invasive, efficient, safe, and effective technologies have emerged to both diagnose and treat this condition. However, even with the availability of these advances among the general health-care community for a period of over 20 years, the adoption and use of these modalities has been limited. Historic difficulty in diagnosing bronchiectasis coupled with the lack of readily available effective treatment certainly contributed to the general lack of interest in this disease as did the misperception that bronchiectasis is uncommon.^4^

Not surprisingly then, it is widely believed that while increasing use of HRCT has led to a marked rise in the number of persons with diagnosed bronchiectasis in current US clinical practice, the disorder remains substantially underdiagnosed, especially among chronic obstructive pulmonary disease (COPD) patients. Estimates of the prevalence of bronchiectasis within COPD vary widely across studies,^2,5–16^ and underdiagnosis of these cases can lead to significant delays in instituting appropriate therapy.^17^

Current clinical management is typically multifaceted and includes treatment of concurrent etiologic conditions, promotion of airway clearance (e.g., mobilization and clearance of secretions), reduction in bronchial inflammation, and suppression or prevention of chronic bronchial infection.^18^ While effective management can interrupt the vicious cycle of bronchiectasis, complications frequently occur. Consequently, persons with bronchiectasis often require intensive and costly medical treatment over extended periods of time.^19^

Notwithstanding the seriousness of the disorder, surprisingly little is known about the current epidemiology of bronchiectasis among US adults, as the limited data that have been reported are now over a decade old and are therefore not reflective of today’s practice patterns, especially use of HRCT as a diagnostic aid.^19,20^ A more recent UK study found that the prevalence of bronchiectasis is “surprisingly common” and has been growing steadily since 2004.^21^ A similar study in Korea also found a higher than expected prevalence of bronchiectasis.^22^ Other studies are older, based on smaller groups, and may not be generalizable to the US population.^23–27^ Because of the limitations of published epidemiological research, a new study using a large health-care claims repository was undertaken. While lacking certain elements of clinical richness, such repositories provide access to the health profile and health-care experience—across the continuum of care settings—of tens of millions of persons over a multiyear period of time and thus contain information on large numbers of patients with specific diagnoses.

## Study objectives

The principal objectives of this research were to estimate the total number of adults with bronchiectasis (noncystic fibrosis), and the total number of adults with newly diagnosed bronchiectasis (non-cystic fibrosis), in current US clinical practice, on an overall basis and within age- and sex-specific subgroups defined therein.

## Methods

### Study design and data source

This study employed a retrospective design and deidentified data from the Truven Health Analytics MarketScan^®^ Commercial Claims and Encounters and Medicare Supplemental and Coordination of Benefits databases (hereinafter, the “MarketScan Database”), a large health-care claims repository (2009–2013) that comprises medical (i.e. facility and professional service) and outpatient pharmacy claims from a large number of participating private US health plans. A detailed description of study methods is provided in the online supplement.

### Source and study populations

The source population comprised all persons who were aged ≥18 years and had comprehensive health benefits for ≥1 day during 2013 (i.e. the referent year). From the source population, all persons who had evidence of bronchiectasis during 2009–2013 were identified based on ≥2 ambulatory encounters with a diagnosis of bronchiectasis (ICD-9-CM 494.x) and dates of service ≥30 days apart; one ambulatory encounter with a diagnosis of bronchiectasis and computed tomography (CT) scan of the thorax (CPT-4 71250, 71260, 71270) within 60 days prior to the encounter or ≥1 hospitalization with a principal or secondary diagnosis of bronchiectasis. Persons who were first flagged as having bronchiectasis in 2013, and who were continuously eligible for comprehensive health benefits for ≥1 year prior to the date of the first such encounter, were designated as having “newly diagnosed” (i.e. incident) disease. Persons who had evidence of bronchiectasis and cystic fibrosis were excluded from the prevalent and incident populations. Case-ascertainment algorithms were largely consistent with methods employed in prior research.^19,28^

Two alternative case-ascertainment algorithms were also employed to evaluate the sensitivity of study results. With the first—which was designed to provide an upper bound on the estimated prevalence of diagnosed bronchiectasis—all persons in the source population who had ≥1 encounters with a diagnosis of bronchiectasis at any time during 2009–2013 were designated as having prevalent disease. The second—employed for purposes of comparison with prior research—required ≥1 diagnosis during the referent year.^20^

### Analyses

Disease prevalence was calculated for each age- and sex-specific stratum by dividing the total number of persons with bronchiectasis in 2013 by the total number of persons in the source population (i.e. persons who had at least one day of eligibility for comprehensive health benefits in CY2013). Disease incidence (annual) was calculated for each age- and sex-specific stratum by dividing the total number of persons with newly diagnosed bronchiectasis in 2013 by the total number of persons in the source population who were continuously eligible for comprehensive health benefits in 2013. Estimated rates of disease prevalence and incidence in the United States were calculated by combining age- and sex-specific rates from the analyses described earlier with corresponding population weights from the US Census.^29^

## Results

### Prevalence

The source population for calculation of disease prevalence included a total of 33.2 million adults aged ≥18 years who had comprehensive health benefits for ≥1 day during 2013 (online supplement - Table 1). Among these persons, 31,122 met our criteria for having evidence of bronchiectasis in 2013 and thus qualified for inclusion in the corresponding study population. Mean age of persons with bronchiectasis was 68 (±14) years, and approximately two-thirds of subjects were female (online supplement—Table 2). The most common comorbidities were COPD, 52%; acute bronchitis, 38%; postinflammatory fibrosis, 14%; and genetic disorders, 10%.

**Figure 1.**
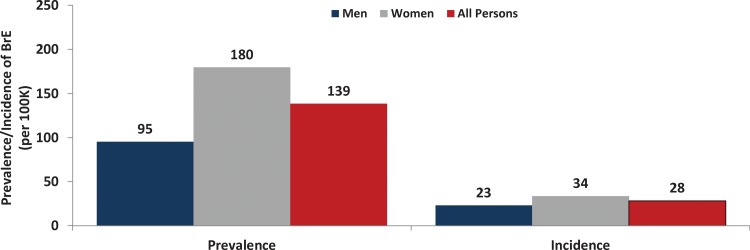
Prevalence and incidence (annual) of bronchiectasis among US adults, overall and by sex.

**Figure 2.**
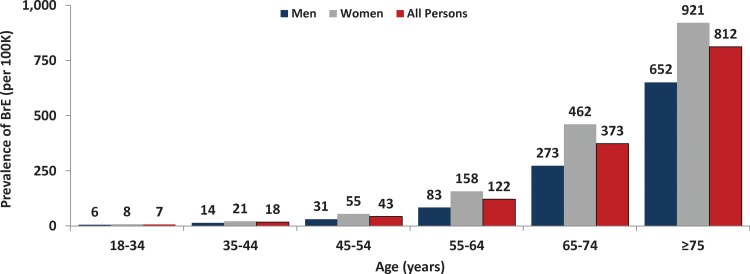
Prevalence of bronchiectasis among US adults, by age and sex.

The overall prevalence of bronchiectasis was estimated to be 139 cases per 100,000 US adults aged ≥18 years and was estimated to be higher for women (180 per 100,000) versus men (95 per 100,000) (Figure 1, online supplement—Table 3). The prevalence of bronchiectasis increased substantially with age, from 7 per 100,000 persons aged 18–34 years to 812 per 100,000 persons aged ≥75 years (Figure 2). Prevalence was higher among women than men at all ages (by 1.3– 1.9 times). The prevalence in US seniors (i.e., adults aged ≥65 years) was 562 cases per 100,000.

### Incidence

The source population for calculation of disease incidence included a total of 23.7 million adults aged ≥18 years who had comprehensive health benefits during all of 2013. Among these persons, 7736 met our criteria for having evidence of newly diagnosed bronchiectasis in 2013, and among these persons, 5120 were also continuously eligible for health benefits for ≥1 year prior to their first evidence of bronchiectasis.

**Figure 3.**
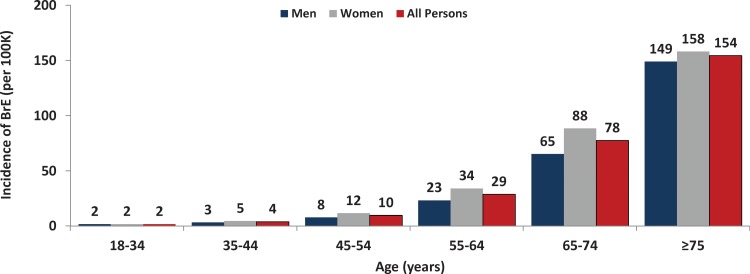
Incidence (annual) of bronchiectasis among US adults, by age and sex.

The overall annual incidence of bronchiectasis was estimated to be 29 cases per 100,000 US adults aged ≥18 years and was estimated to be higher for women (34 per 100,000) versus men (23 per 100,000) (Figure 1, online supplement—Table 4). The incidence of bronchiectasis increased substantially with age, from 2 per 100,000 persons aged 18–34 years to 154 per 100,000 persons aged ≥75 years (Figure 3). Incidence was higher among women than men at all ages (by 1.1–1.5 times).

### Sensitivity analyses

When employing the broader case-ascertainment algorithm requiring ≥1 diagnosis of bronchiectasis at any time during the study period (i.e. 2009–2013), age- and sex-specific estimates of disease prevalence were 39–128% higher versus corresponding base case values (online supplement - Table 5), and overall estimated disease prevalence in the United States was 213 per 100,000 persons. When employing the Seitz case-ascertainment algorithm requiring ≥1 diagnosis of bronchiectasis during the referent year (i.e. 2013), age- and sex-specific estimates of disease prevalence varied by ±1–28% versus corresponding base case values (online supplement—Table 6), and overall estimated disease prevalence in the United States was 110 per 100,000 persons.

## Discussion

**Figure 4.**
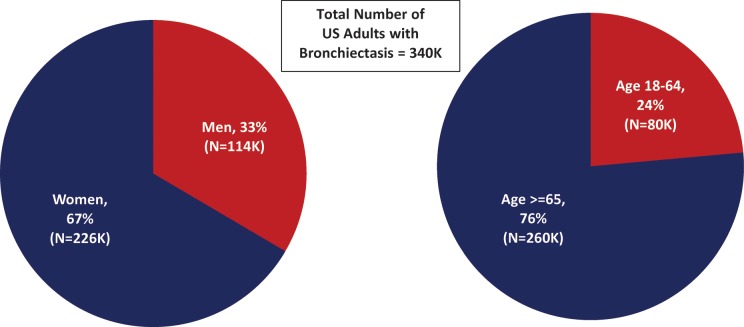
Number of US adults with bronchiectasis, by age and sex.

Using a retrospective cohort design and data from a large health-care claims repository, we undertook an evaluation to better understand the current epidemiology of noncystic fibrosis bronchiectasis among US adults of all ages. The findings of this evaluation suggest that the number of adults with bronchiectasis in the United States is substantially higher than previously believed, with an estimated 340,000 (and as many as 522,000) US adults receiving treatment for bronchiectasis in the United States each year, most of whom are women (67%, or 226,000) and most of whom are aged ≥65 years (76% or 260,000) (Figure 4).

In the 2005 study by Weycker et al.^19^ which utilized a private health-care claims database (1999–2001) and similar case-ascertainment algorithms, the prevalence of bronchiectasis was reported to be 52.3 cases per 100,000 US adults aged ≥18 years, corresponding to an estimated 110,000 adults of all ages with bronchiectasis in the United States. In the 2012 study by Seitz et al.^20^ which utilized Medicare outpatient claims data and an algorithm requiring a single diagnosis in a given year, disease prevalence was reported to increase substantially from 2000 to 2007 (8.7% annual growth rate), from 223 to 388 per 100,000 men aged ≥65 years and from 322 to 553 per 100,000 women aged ≥65 years; the authors estimated that there were >190,000 US adults aged ≥65 years with bronchiectasis in 2007. By comparing the results from Weycker et al. and those from the present study, it appears that the marked increase over time in disease prevalence reported by Seitz et al. has continued: An almost identical 8% from 2001 (52 per 100,000) to 2013 (139 per 100,000) among US adults of all ages. While the precise reasons underlying the increasing prevalence of diagnosed bronchiectasis are unknown, it may be explained—at least in part—by wider/better surveillance, changing population demographics, and/or an underlying increase in morbidity.

Although the etiology of bronchiectasis is unknown in many cases, there is growing awareness regarding the importance of COPD as an underlying cause. In older studies, COPD was not mentioned as a possible cause of bronchiectasis.^27,30^ While it is not possible in the present study to determine the underlying cause of bronchiectasis, we note that 52% of patients with bronchiectasis had comorbid COPD, further highlighting the evolving diagnostic philosophy of this condition.

As noted earlier, there are reasons to believe that bronchiectasis remains significantly underdiagnosed in clinical practice, particularly among COPD patients. In the study by Tilert et al., 5.4% of the US population aged 40–79 years old was reported to have GOLD stage II–IV (moderate to very severe) COPD—including diagnosed and undiagnosed cases—or about 7.7 million persons in total.^31^ While the prevalence of bronchiectasis in patients with COPD has been documented in various sources,^7,9,12,13,16,32^ a recent meta-analysis reported that 54.3% of COPD patients also had bronchiectasis.^14^ Using a weighted average of estimates from these sources, we project that about 4.2 million US adults over 40 years old may have bronchiectasis in the United States, suggesting that there is a large pool of patients with undiagnosed disease. Despite the widening use of advanced diagnostic tools such as HRCT, the clinical and economic burden of undiagnosed bronchiectasis is a cause for great concern. Clearly, more needs to be done to promote additional research in this area and to disseminate findings to the broadest possible audience.

Based on the findings of this study, it appears that the prevalence of diagnosed bronchiectasis is much higher than previously reported and that the prevalence of undiagnosed cases is undoubtedly still larger. The resulting economic burden to the US health-care system is likely to be substantial, due in particular, to the frequent and costly hospitalizations required by such patients as reported in the studies by Weycker et al. and Seitz et al.^19,33^ With the current shift in health-care economics from the present paradigm of “fee-for-service” to “pay-for-performance” health care, new metrics for performance are emerging from Medicare (Centers for Medicare and Medicaid Services) including, but not limited to, economic penalties associated with 30-day readmission rates for patients with COPD. Recent literature suggests that comorbid bronchiectasis can contribute substantially to the overall morbidity of patients suffering with COPD: more specifically, leading to increased COPD exacerbations, hospitalizations, and presumably readmissions.^34^ This shift in medical economics coupled with the aforementioned advances in the diagnosis and treatment of bronchiectasis will hopefully motivate primary care providers and pulmonary specialists to take more interest in bronchiectasis as a disease state and more actively pursue its diagnosis and treatment. Additional research aimed at updating the economic burden of bronchiectasis among adults of all ages is warranted.

We note that some of the differences between the results from the present study and the two previously published evaluations—Seitz (2012) and Weycker (2005)—may be explained by variation in the data sources (private healthcare claims, Medicare 5% Sample, and private healthcare claims), the calendar years of observation (2009–2013, 2000–2007, 1999–2001), and case-ascertainment algorithms (multifactorial/5-year window, single diagnosis/1-year window, multifactorial/3-year window).^19,20^ We also note, however, that when employing similar algorithms as those used previously, the general conclusion of the present study that the prevalence of bronchiectasis is substantially higher than previously reported remained true.

Certain aspects of our operational definition of bronchiectasis may overestimate disease prevalence and incidence. While our criterion of ≥2 outpatient encounters with diagnoses of bronchiectasis is probably a sensitive measure of case-ascertainment, it may not be sufficiently specific. We note, however, that most subjects flagged as having bronchiectasis in this study had a substantially higher number of encounters with a diagnosis of bronchiectasis (mean [SD] number of encounters = 7.1 [11.1]) during the 5-year period of interest (mean [SD] years of observation = 4.2 [1.3]). Moreover, although HRCT has been shown to be highly accurate in diagnosing bronchiectasis—and is currently the gold standard in this use—ICD-9-CM and CPT-4 procedure codes do not distinguish HRCT from CT with lesser degrees of resolution. We assumed, however, that most persons who underwent CT testing received the recommended HRCT and that any upward bias from using the CT criterion would be small.

On the other hand, there are reasons to believe that, despite deficiencies in the diagnostic criteria that we employed, our estimates of prevalence and incidence are conservative. We could not identify patients with undiagnosed bronchiectasis and those who may have been diagnosed with bronchiectasis but did not have a qualifying encounter during the period of interest. Our case-finding criteria undoubtedly excluded some actual cases of bronchiectasis, particularly milder ones without multiple encounters. For this reason, we also calculated disease prevalence using broad case-finding criteria, which undoubtedly included cases for which bronchiectasis was suspected but not confirmed. We believe that the true prevalence of disease probably falls somewhere between the two estimates. Finally, we note that our study employed data from a convenience—albeit large—sample of persons enrolled in private health insurance programs in the United States. Persons with such insurance may differ systematically from the rest of the US population in terms of their health status and/or health-care experience as well as from persons in other countries. For example, persons without health insurance are more likely to be smokers and to have COPD, which may be associated with the development of bronchiectasis.^35^ Accordingly, caution should be used in generalizing the results of this study to other populations and settings.

In summary, the findings of this study suggest that between 340,000 and 522,000 adults are receiving treatment for bronchiectasis in US clinical practice and that 70,000 adults are newly diagnosed with bronchiectasis each year in the United States. The findings of this study also suggest that—consistent with prior research—the diagnosis of bronchiectasis continues to increase in clinical practice (annual growth rate since 2001, 8%)—presumably due, at least in part, to recent advances in, and increased use of, radiologic techniques—and that diagnosed cases reflect a relatively small segment of the overall bronchiectasis population. Taken collectively, this study indicates that the prevalence and incidence of bronchiectasis is substantially higher than previously reported, underscoring the importance of diligent surveillance and attentive treatment for this condition. Additional research is needed to validate the findings of this study, to identify the reasons for increased prevalence, and to promote education about bronchiectasis nationally.

## Supplementary Material

Supplementary material
