# Supplementary material for: Prevalence and incidence of noncystic fibrosis bronchiectasis among US adults in 2013
Source: Chron Respir Dis. 2017 May 30;14(4):377–84. doi: 10.1177/1479972317709649 (PMC5729734; doi:10.1177/1479972317709649)
Supplement: Supplementary material [file Epidemiology_of_BrE_in_US_Clinical_Practice_v2.pdf]

Online Supplement for Prevalence and Incidence of Non-Cystic Fibrosis Bronchiectasis among US Adults in 2013:
Study Methods

METHODS

Study Design and Data Source

This study employed a retrospective design and data from the Truven Health Analytics MarketScan® Commercial Claims and Encounters (CCAE) and Medicare Supplemental and Coordination of Benefits (MDCR) databases (hereinafter, the "MarketScan Database"). The CCAE database contains data on the health insurance claims of employees of large, self-insured corporations and their dependents, along with data from a few commercial health plans. The MDCR database contains enrollment information, Medicare claims, and employer-sponsored health insurance claims of Medicare-eligible retirees, and includes only plans where both the Medicare-paid amounts and the employer-paid amounts are available and evident on the claims. The data extract for this study was limited to insurers that provided healthcare claims information to the MarketScan Database during the entire 5-year period from 2009-2013.

Data available from each facility and professional-service claim include dates and places of service, diagnoses, procedures performed/services rendered, and quantity of services (professional-service claims only). Data available from each outpatient pharmacy claim include the drug (class) dispensed, dispensing date, quantity dispensed, and number of days supplied. Medical and pharmacy claims also include amounts paid (i.e., reimbursed) by health plans as well as by patients to providers for services rendered. Selected demographic and eligibility information (including age, sex, geographic region of residence, dates of plan eligibility) is available for all health plan enrollees. All data can be arrayed to provide a detailed chronology of medical and pharmacy services used by each plan member over time.

The study extract was de-identified prior to its release to study investigators, as set forth in the corresponding Data Use Agreement. The MarketScan Database has been evaluated and certified by an independent third party to be in compliance with the Health Insurance Portability and Accountability Act (HIPAA) of 1996 statistical de-identification standards and to satisfy the conditions set forth in Sections 164.514 (a)-(b)1ii of the HIPAA Privacy Rule regarding the determination and documentation of statistically de-identified data. Use of the study extract for health services research is therefore fully compliant with the HIPAA Privacy Rule.

Source and Study Populations

The source population comprised all persons who were aged ≥18 years and had comprehensive health benefits for ≥1 day during 2013 (i.e., the referent year). From the source population, all persons who had evidence of bronchiectasis during 2009-2013 were identified based on ≥2 ambulatory encounters with a diagnosis of bronchiectasis (ICD-9-CM 494.x) and dates of service ≥30 days apart; one ambulatory encounter with a diagnosis of bronchiectasis, and computed tomography (CT) scan of the thorax (CPT-4 71250, 71260, 71270) within 60 days prior to the encounter; or ≥1 hospitalization with a principal or secondary diagnosis of bronchiectasis.

Persons who were first flagged as having bronchiectasis in 2013, and who were continuously eligible for comprehensive health benefits for ≥1 year prior to the date of the first such encounter, were designated as having "newly diagnosed" (i.e., incident) disease. Encounters for "ancillary" services (e.g., laboratory assessments) that may be used to assist in the diagnosis of diseases were excluded from consideration in identifying the source population. Persons who had evidence of bronchiectasis and cystic fibrosis were excluded from the prevalent and incident populations. Case-ascertainment algorithms were largely consistent with methods employed in prior research.[7,9]

Two alternative case-ascertainment algorithms were also employed to evaluate the sensitivity of study results. With the first—which was designed to provide an upper bound on the estimated prevalence of diagnosed bronchiectasis—all persons in the source population who had ≥1 encounters with a diagnosis of bronchiectasis at any time during 2009-2013 were designated as having prevalent disease. The second—employed for purposes of comparison with prior research—required ≥1 diagnosis during the referent year.[8]

Analyses

Demographic and clinical characteristics of persons with prevalent and incident (diagnosed) bronchiectasis were descriptively analyzed. The presence of selected comorbidities was identified based on ≥2 ambulatory encounters (≥30 days apart) or ≥1 hospitalization with a corresponding diagnosis during the two-year period beginning on January 1, 2012 and ending on December 31, 2013.

Disease prevalence was calculated for each age- and sex-specific stratum by dividing the total number of persons with bronchiectasis in 2013 by the total number of persons in the source population (i.e., persons who had at least one day of eligibility for comprehensive health benefits in CY2013). Disease incidence (annual) was calculated for each age- and sex-specific stratum by dividing the total number of persons with newly diagnosed bronchiectasis in 2013 by the total number of persons in the source population who were continuously eligible for comprehensive health benefits in 2013. Confidence intervals for study measures were not reported due to the large sample size and corresponding small standard errors.

Estimated rates of disease prevalence and incidence in the US were calculated by combining age- and sex-specific rates from the analyses described above with corresponding population weights from the US Census.[10] Sensitivity analyses were conducted considering each of the alternative case-ascertainment algorithms, as described above.

Online Supplement — Table 1. Selection of source and study populations

Inclusion Criteria	No. of Persons
Prevalence	
Source Population	
All persons aged ≥18 years with ≥1 day of comprehensive health benefits during year of interest	33,204,504
Study Population	
Persons with BrE in year of interest	31,122
≥2 ambulatory encounters with diagnosis of BrE separated by ≥30 days; OR	21,284
1 ambulatory encounter with diagnosis of BrE and CT scan within 60 days; OR	15,657
≥1 hospitalization with 1° or 2° diagnosis of BrE	8,975
Incidence	
Source Population	
All persons aged ≥18 years continuously eligible for comprehensive health benefits in year of interest	23,722,415
Study Population	
Persons with Newly Diagnosed BrE in year of interest	7,482
≥2 ambulatory encounters with diagnosis of BrE separated by ≥30 days; OR	3,711
1 ambulatory encounter with diagnosis of BrE and CT scan within 60 days; OR	3,806
≥1 hospitalization with 1° or 2° diagnosis of BrE	2,231
Plus continuously eligible for comprehensive health benefits for ≥1 year prior first evidence of BrE	5,120

BrE: bronchiectasis; CT: computed tomography

*All available data during CY2009-CY2013 were used to identify persons with BrE in CY2013

Online Supplement — Table 2. Demographic characteristics and clinical profile of persons with BrE and persons newly diagnosed with BrE

	Persons with BrE			
	All (N=31,122)	Excluding Newly Diagnosed (N=26,002)	Persons Newly Diagnosed with BrE (N=5,120)	p-value*
Patient Characteristics				
Age (years)				
Mean (SD)	68.2 (14.4)	68.3 (14.4)	67.7 (14.1)	0.004
Median	69	70	69	
Age Group, years, n (%)				
18-34	765 (2.5)	646 (2.5)	119 (2.3)	0.059
35-44	1,102 (3.5)	928 (3.6)	174 (3.4)	
45-54	2,979 (9.6)	2,470 (9.5)	509 (9.9)	
55-64	7,438 (23.9)	6,142 (23.6)	1,296 (25.3)	
65-74	7,107 (22.8)	5,890 (22.7)	1,217 (23.8)	
≥75	11,731 (37.7)	9,926 (38.2)	1,805 (35.3)	
Gender, n (%)				
Female	20,482 (65.8)	17,401 (66.9)	3,081 (60.2)	<0.001
Male	10,640 (34.2)	8,601 (33.1)	2,039 (39.8)	
Geographic Region, n (%)				
Midwest	7,308 (23.5)	5,925 (22.8)	1,383 (27.0)	<0.001
South	8,867 (28.5)	7,366 (28.3)	1,501 (29.3)	
Northeast	6,517 (20.9)	5,597 (21.5)	920 (18.0)	
West	7,669 (24.6)	6,475 (24.9)	1,194 (23.3)	
Unknown	761 (2.4)	639 (2.5)	122 (2.4)	
Payer Type, n (%)				
HMO	1,829 (5.9)	1,532 (5.9)	297 (5.8)	0.051
PPO	7,146 (23.0)	5,921 (22.8)	1,225 (23.9)	
POS	957 (3.1)	787 (3.0)	170 (3.3)	
Comprehensive	484 (1.6)	384 (1.5)	100 (2.0)	
Medicare Risk	19,054 (61.2)	15,984 (61.5)	3,070 (60.0)	
Other	1,482 (4.8)	1,249 (4.8)	233 (4.6)	
Unknown	170 (0.5)	145 (0.6)	25 (0.5)	
Clinical Profile				
Comorbidities, n (%)				
Acute Bronchitis	11,657 (37.5)	9,394 (36.1)	2,263 (44.2)	<0.001
COPD	16,017 (51.5)	13,175 (50.7)	2,842 (55.5)	<0.001
Genetic Disorders**	3,146 (10.1)	2,771 (10.7)	375 (7.3)	<0.001
Inflammatory Bowel Disease	695 (2.2)	574 (2.2)	121 (2.4)	0.491
Lung Malignancies	1,204 (3.9)	920 (3.5)	284 (5.5)	<0.001
Post-Inflammatory Pulmonary Fibrosis	4,473 (14.4)	3,470 (13.3)	1,003 (19.6)	<0.001
Pulmonary Nontuberculosis Mycobacterial Disease	1,988 (6.4)	1,747 (6.7)	241 (4.7)	<0.001
Rheumatoid Disease	2,316 (7.4)	1,943 (7.5)	373 (7.3)	0.641
Evidence of Use of, n (%)				
High Freq. Chest Wall Oscillation Air-Pulse System	740 (2.4)	643 (2.5)	97 (1.9)	0.013
Electric/Pneumatic Percussor	8 (0.0)	8 (0.0)	0 (0.0)	0.209
Oscillatory Positive Expiratory Pressure Device	510 (1.6)	411 (1.6)	99 (1.9)	0.069
Respiratory Suction Pump	263 (0.8)	214 (0.8)	49 (1.0)	0.338
Cough Stimulating Device	11 (0.0)	7 (0.0)	4 (0.1)	0.075
Nebulizer Compressor	5,492 (17.6)	4,441 (17.1)	1,051 (20.5)	<0.001
Bronchoscopy	4,492 (14.4)	3,263 (12.5)	1,229 (24.0)	<0.001
Supplemental Oxygen	7,327 (23.5)	6,004 (23.1)	1,323 (25.8)	<0.001

BrE: bronchiectasis; COPD: chronic obstructive pulmonary disease

*Test for differences between persons with BrE (excluding newly diagnosed) and persons newly diagnosed with BrE

**Situs inversus, common variable immunodeficiency, IgG deficiency, allergic bronchopulmonary aspergilliosis, and congenital bronchiectasi

Online Supplement — Table 3. Prevalence of bronchiectasis among US adults

	Study Database			US (2013)		
		Persons with BrE				
	Population[†]	n	Rate per 100K	No. of Persons	No. of Persons with BrE	Rate of BrE per 100K
All Persons	33,204,504	31,122	93.7	245,273,438	339,843	138.6
Age						
18-34	10,744,007	765	7.1	74,980,662	5,312	7.1
35-44	6,191,587	1,102	17.8	40,513,133	7,160	17.7
45-54	6,830,999	2,979	43.6	43,458,851	18,772	43.2
55-64	6,066,557	7,438	122.6	40,077,581	48,838	121.9
≥65	3,371,354	18,838	558.8	46,243,211	259,761	561.7
65-74	*1,919,126*	*7,107*	*370.3*	*26,398,290*	*98,586*	*373.5*
≥75	*1,452,228*	*11,731*	*807.8*	*19,844,921*	*161,175*	*812.2*
Men	15,884,532	10,640	67.0	119,352,940	113,763	95.3
Age						
18-34	5,255,785	323	6.1	38,095,493	2,341	6.1
35-44	2,975,605	417	14.0	20,159,229	2,825	14.0
45-54	3,250,461	1,006	30.9	21,425,044	6,631	30.9
55-64	2,863,808	2,385	83.3	19,321,882	16,091	83.3
≥65	1,538,873	6,509	423.0	20,351,292	85,874	422.0
65-74	*929,665*	*2,540*	*273.2*	*12,349,045*	*33,740*	*273.2*
≥75	*609,208*	*3,969*	*651.5*	*8,002,247*	*52,135*	*651.5*
Women	17,319,972	20,482	118.3	125,920,498	226,080	179.5
Age						
18-34	5,488,222	442	8.1	36,885,169	2,971	8.1
35-44	3,215,982	685	21.3	20,353,904	4,335	21.3
45-54	3,580,538	1,973	55.1	22,033,807	12,141	55.1
55-64	3,202,749	5,053	157.8	20,755,699	32,746	157.8
≥65	1,832,481	12,329	672.8	25,891,919	173,886	671.6
65-74	*989,461*	*4,567*	*461.6*	*14,049,245*	*64,846*	*461.6*
≥75	*843,020*	*7,762*	*920.7*	*11,842,674*	*109,040*	*920.7*

BrE: bronchiectasis

[†]Number of persons with at least one day of eligibility for health benefits in CY2013

Online Supplement — Table 4. Incidence of bronchiectasis among US adults

	Study Database			US (2013)		
		Persons with Newly Diagnosed BrE				
	Population†	n	Rate per 100K	No. of Persons	No. of Persons with New BrE	Rate of New BrE per 100K
All Persons	23,722,415	5,120	21.6	245,273,438	69,790	28.5
Age						
18-34	6,866,011	119	1.7	74,980,662	1,298	1.7
35-44	4,420,834	174	3.9	40,513,133	1,583	3.9
45-54	5,222,339	509	9.7	43,458,851	4,202	9.7
55-64	4,467,087	1,296	29.0	40,077,581	11,573	28.9
≥65	2,746,144	3,022	110.0	46,243,211	51,134	110.6
65-74	*1,575,860*	*1,217*	*77.2*	*26,398,290*	*20,491*	*77.6*
≥75	*1,170,284*	*1,805*	*154.2*	*19,844,921*	*30,643*	*154.4*
Men	11,277,336	2,039	18.1	119,352,940	27,434	23.0
Age						
18-34	3,351,789	56	1.7	38,095,493	636	1.7
35-44	2,102,936	69	3.3	20,159,229	661	3.3
45-54	2,466,626	191	7.7	21,425,044	1,659	7.7
55-64	2,097,735	488	23.3	19,321,882	4,495	23.3
≥65	1,258,250	1,235	98.2	20,351,292	19,983	98.2
65-74	*764,040*	*499*	*65.3*	*12,349,045*	*8,065*	*65.3*
≥75	*494,210*	*736*	*148.9*	*8,002,247*	*11,917*	*148.9*
Women	12,445,079	3,081	24.8	125,920,498	42,355	33.6
Age						
18-34	3,514,222	63	1.8	36,885,169	661	1.8
35-44	2,317,898	105	4.5	20,353,904	922	4.5
45-54	2,755,713	318	11.5	22,033,807	2,543	11.5
55-64	2,369,352	808	34.1	20,755,699	7,078	34.1
≥65	1,487,894	1,787	120.1	25,891,919	31,151	120.3
65-74	*811,820*	*718*	*88.4*	*14,049,245*	*12,426*	*88.4*
≥75	*676,074*	*1,069*	*158.1*	*11,842,674*	*18,725*	*158.1*

BrE: bronchiectasis; Dx: diagnosed

†Number of persons continuously eligible for health benefits in CY2013

Online Supplement — Table 5. Prevalence of bronchiectasis among US adults (based on >=1 diagnosis code anytime from 2009-2013)

	Study Database			US (2013)		
		Persons with BrE				
	Population†	n	Rate per 100K	No. of Persons	No. of Persons with BrE	Rate of BrE per 100K
All Persons	33,204,504	49,214	148.2	245,273,438	521,579	212.7
Age						
18-34	10,744,007	1,733	16.1	74,980,662	12,029	16.0
35-44	6,191,587	2,327	37.6	40,513,133	15,135	37.4
45-54	6,830,999	5,477	80.2	43,458,851	34,540	79.5
55-64	6,066,557	12,026	198.2	40,077,581	79,031	197.2
≥65	3,371,354	27,651	820.2	46,243,211	380,845	823.6
65-74	*1,919,126*	*10,841*	*564.9*	*26,398,290*	*150,180*	*568.9*
≥75	*1,452,228*	*16,810*	*1,157.5*	*19,844,921*	*230,665*	*1,162.3*
Men	15,884,532	17,764	111.8	119,352,940	185,093	155.1
Age						
18-34	5,255,785	724	13.8	38,095,493	5,248	13.8
35-44	2,975,605	914	30.7	20,159,229	6,192	30.7
45-54	3,250,461	1,909	58.7	21,425,044	12,583	58.7
55-64	2,863,808	4,114	143.7	19,321,882	27,757	143.7
≥65	1,538,873	10,103	656.5	20,351,292	133,313	655.1
65-74	*929,665*	*4,096*	*440.6*	*12,349,045*	*54,409*	*440.6*
≥75	*609,208*	*6,007*	*986.0*	*8,002,247*	*78,905*	*986.0*
Women	17,319,972	31,450	181.6	125,920,498	336,486	267.2
Age						
18-34	5,488,222	1,009	18.4	36,885,169	6,781	18.4
35-44	3,215,982	1,413	43.9	20,353,904	8,943	43.9
45-54	3,580,538	3,568	99.6	22,033,807	21,957	99.6
55-64	3,202,749	7,912	247.0	20,755,699	51,274	247.0
≥65	1,832,481	17,548	957.6	25,891,919	247,531	956.0
65-74	*989,461*	*6,745*	*681.7*	*14,049,245*	*95,771*	*681.7*
≥75	*843,020*	*10,803*	*1,281.5*	*11,842,674*	*151,760*	*1,281.5*

BrE: bronchiectasis

†Number of persons with at least one day of eligibility for health benefits in CY2013

Online Supplement — Table 6. Prevalence of bronchiectasis among US adults (based on >=1 diagnosis code in 2013)

	Study Database			US (2013)		
		Persons with BrE				
	Population†	n	Rate per 100K	No. of Persons	No. of Persons with BrE	Rate of BrE per 100K
All Persons	33,204,504	25,088	75.6	245,273,438	268,958	109.7
Age						
18-34	10,744,007	793	7.4	74,980,662	5,508	7.3
35-44	6,191,587	1,059	17.1	40,513,133	6,891	17.0
45-54	6,830,999	2,645	38.7	43,458,851	16,671	38.4
55-64	6,066,557	6,098	100.5	40,077,581	40,046	99.9
≥65	3,371,354	14,493	429.9	46,243,211	199,841	432.2
65-74	*1,919,126*	*5,893*	*307.1*	*26,398,290*	*81,738*	*309.6*
≥75	*1,452,228*	*8,600*	*592.2*	*19,844,921*	*118,103*	*595.1*
Men	15,884,532	8,729	55.0	119,352,940	91,727	76.9
Age						
18-34	5,255,785	338	6.4	38,095,493	2,450	6.4
35-44	2,975,605	423	14.2	20,159,229	2,866	14.2
45-54	3,250,461	902	27.7	21,425,044	5,945	27.7
55-64	2,863,808	1,982	69.2	19,321,882	13,372	69.2
≥65	1,538,873	5,084	330.4	20,351,292	67,094	329.7
65-74	*929,665*	*2,115*	*227.5*	*12,349,045*	*28,094*	*227.5*
≥75	*609,208*	*2,969*	*487.4*	*8,002,247*	*38,999*	*487.4*
omen	17,319,972	16,359	94.5	125,920,498	177,231	140.7
Age						
18-34	5,488,222	455	8.3	36,885,169	3,058	8.3
35-44	3,215,982	636	19.8	20,353,904	4,025	19.8
45-54	3,580,538	1,743	48.7	22,033,807	10,726	48.7
55-64	3,202,749	4,116	128.5	20,755,699	26,674	128.5
≥65	1,832,481	9,409	513.5	25,891,919	132,747	512.7
65-74	*989,461*	*3,778*	*381.8*	*14,049,245*	*53,643*	*381.8*
≥75	*843,020*	*5,631*	*668.0*	*11,842,674*	*79,104*	*668.0*

BrE: bronchiectasis

†Number of persons with at least one day of eligibility for health benefits in CY2013
